# Impact of continuity of care under the model of healthcare integration on treatment outcomes and prognostic recovery of orthodontic patients

**DOI:** 10.3389/fmed.2025.1632725

**Published:** 2025-11-10

**Authors:** Qing Li, Ruihan Wu, Chen Du, Qifeng Miao

**Affiliations:** Department of Stomatology, Xuzhou Central Hospital, Xuzhou, Jiangsu, China

**Keywords:** complications, continuity of care, healthcare integration, mental health, orthodontic, treatment outcomes

## Abstract

Malocclusion is one of the most serious oral problems following dental caries and periodontal disease. Although orthodontic treatment can effectively improve these problems, improper care often leads to poor orthodontic results or complications such as gingivitis. This is because the treatment period is long. Based on this, this study aimed to investigate the effect of continuity of care under the integrated model of health care on the treatment efficacy and prognosis of orthodontic patients. This study used a randomized controlled design. From June 2022 to December 2022, 68 patients who received orthodontic treatment in our hospital were selected as the control group, and from January 2023 to May 2023, 68 patients were selected as the intervention group. The control group received routine care, while the intervention group received continuity of care under the integrated care model, including the establishment of an integrated care team, training team members, psychological care, dietary care, oral care, health education and regular follow-up. The results of the study showed that the plaque index (PLI) and gingival index (GI) of the intervention group were significantly better than those of the control group. The incidence of complications in the intervention group was lower than that in the control group. In addition, compared with the control group, the intervention group also showed significant improvement in self-management ability, compliance and psychological health. The nursing satisfaction of the intervention group was higher than that of the control group. In conclusion, continuity of care under the model of healthcare integration can significantly improve the periodontal health status of orthodontic patients, enhance their self-management ability and psychological health, reduce the occurrence of complications, and increase nursing satisfaction.

## 1 Introduction

Malocclusion is one of the most serious oral problems after dental caries and periodontal disease. According to statistics, the prevalence of malocclusion among children and adolescents ranges from 39 to 93% (1, 2). In China, the prevalence of malocclusion is about 91.20%, which not only seriously affects the esthetic appearance of the teeth, but also poses greater threats to periodontal health, mental health and physical health (3). The high prevalence indicates high demand, and more and more patients are beginning to receive orthodontic treatment (4). Orthodontics refers to the process of repairing problems such as uneven tooth arrangement and abnormal tooth shape through oral techniques, which can help patients restore good chewing function and tooth esthetics (5). However, clinical orthodontic treatment often takes a long time, with an average treatment period of 2 to 3 years. Poor orthodontic care can easily lead to poor orthodontic results or even complications such as dental caries and periodontitis (6). Therefore, improving patients’ knowledge of orthodontic-related care, compliance and oral care ability will directly affect the outcome of orthodontic treatment.

The healthcare integration model refers to a process where doctors and nurses with certain professional knowledge and skills, under the conditions of equality, respect, and mutual trust, provide medical care services to patients through communication, coordination, and joint decision-making (7). The clear division of labor between doctors and nurses is also a widely management model in domestic hospitals (8), which can effectively improve the efficiency of treatment and care and better achieve the goal of patient-centered care. On the other hand, continuity of care is a systematic and individualized care aimed at providing comprehensive and continuous care to patients during the process of health recovery (9). For orthodontic treatment, continuity of care is very necessary. Providing health education for orthodontic patients is a common nursing approach, which can enhance patients’ awareness and ability of oral care, thereby improving the effect of orthodontic treatment and reducing the occurrence of complications.

Based on this, the present study aimed to investigate the effects of continuity of care under the integrated model of health care on the orthodontic effect and prognosis of orthodontic patients, and to comprehensively assess the applicability of this model in orthodontic patients.

## 2 Methods

### 2.1 Study design

This was a randomized controlled study aimed at exploring the effect of continuity of care under the integrated model of health care on orthodontic effect and prognosis of orthodontic patients, and providing pilot research and data support for effective care strategies for orthodontic patients. The study was approved by the Ethics Committee of the hospital, and all applied subjects signed an informed consent form before enrolment.

### 2.2 Participants

By using the random sampling method, patients who received orthodontic treatment in our hospital from June 2022 to December 2022 were selected as the control group. Patients who underwent orthodontic surgery and received postoperative continuous care in our hospital from January 2023 to May 2023 were selected as the intervention group. In the selection of orthodontic appliances, only one type of orthodontic appliance was used, namely the fixed buccal braces.

Inclusion criteria: (1) Received orthodontic treatment in our hospital; (2) Received postoperative continuity of care.

Exclusion criteria: (1) With severe caries, periodontal disease; (2) With severe organic disease; (3) With mental disease or cognitive impairment.

### 2.3 Sample size calculation

The sample size was calculated using PASS 11, with *α* value set as 0.05 and 1-*β* value set as 0.9. The calculated sample size was 60 cases. Considering a 10% dropout rate, each group should have no fewer than 66 cases. In this study, the number of cases in each group was determined to be 68, and the total number of cases for both groups was 136.

### 2.4 Intervention

The control group was given routine care, which consisted of routine health education and telephone follow-up. At the end of orthodontic treatment, the nursing staff provided one-on-one health education, covering aspects such as oral care, pain management, and life precautions. Patients were required to attend follow-up visits on time and go to the hospital for follow-up if any abnormalities occurred. Patients were asked to have a telephone follow-up every 2 weeks and a routine check-up every 2 months to monitor their recovery.

The intervention group was given continuity of care under the integrated healthcare model.

1. Establishment of medical and nursing care team: The chief dentist of our hospital served as the team leader, responsible for providing clinical knowledge guidance; the head nurse of the stomatology department acted as the deputy team leader, responsible for organizing the training of the team members and supervising the quality of care; 10 oral nurses were team members, responsible for implementing specific nursing tasks.
2. Team member training: The head nurses of the hospital organized team members for training. The training covered professional knowledge of oral care and guidance on nursing skills, and they established unified orthodontic care standards. Before officially starting the nursing work, the team members passed the pre-job assessment.
3. Implementation of continuity of care: Nurse established dental orthodontic treatment records for patients, which included the patient’s oral diagnosis, oral condition, as well as care suggestions and instructions.
  - Psychological care: Patients usually worry about the pain caused by orthodontic treatment, the inconvenience of wearing orthodontic appliances, and the recovery effect. Team members used this as the entry point for psychological care, answering patients’ questions and concerns about psychological issues, and striving to alleviate patients’ negative psychological reactions. They introduced the treatment methods and possible adverse reactions in detail, enabling patients to accept orthodontic treatment with a good mindset.
  - Dietary care: During orthodontic treatment, patients were warned to avoid consuming hard, sticky, spicy, and greasy foods, and to reduce the intake of snacks and candies.
  - Oral care: Patients were required to brush their teeth after each meal. First, they brushed the orthodontic device horizontally, and then used the Becton method, brushing for at least 5 min each time. After brushing, they checked in the mirror if there were food residues between the teeth and around the brackets. If so, they used a dental interdental brush for cleaning. If conditions did not permit, they rinsed their mouth with water.
  - Health education: Medical staff invited patients to join the orthodontic continuous care WeChat group. Medical staff used the health education resources of the medical association to regularly post health knowledge related to orthodontics in the WeChat group in the form of text, pictures, and videos, to enhance patients’ and their families’ understanding of orthodontic care. On a fixed day each week, dental department lecturers conducted health education lectures for orthodontic treatment patients, covering daily oral hygiene maintenance, dietary guidance, and psychological care. After the lectures, the patients were organized to communicate with each other, allowing the medical staff to understand the psychological state during the orthodontic treatment process and the problems they encountered in daily life, and answer their questions.
  - Follow-up: During the patient’s stay at home, the medical staff conducted a weekly follow-up call to monitor the patient’s daily oral care at home. A monthly follow-up was conducted, and a personalized oral hygiene, daily diet and follow-up schedule were formulated based on the patient’s specific condition. To ensure the accuracy and reliability of the research, we conducted a strict statistical analysis of the attendance rates for hospital visits and home visits. Regarding hospital visits, patients were required to return to the hospital for re-examination on time and to be followed up promptly in case of any abnormalities. The overall attendance rate for hospital visits reached 100%. In home visits, the attendance rate for telephone follow-ups was 100% and the attendance rate for monthly face-to-face visits was 100%.

Both groups underwent intervention measures for a period of 6 months.

### 2.5 Outcomes

1. Before the intervention, 3 months after the intervention, and 6 months after the intervention, the gingival index (GI) and plaque index (PLI) were used to evaluate the periodontal health status (10). The evaluation criteria for the GI: 0 points for healthy gums; 1 point for slight changes in gum color and mild swelling without bleeding upon probing; 2 points for red gums with a luster and swelling, accompanied by bleeding upon probing; 3 points for spontaneous bleeding tendency, significantly red, swollen or ulcerated gums. The evaluation criteria for the PI: 0 points for no plaque; 1 point for detectable adherent membrane at the gum margins and neck regions upon probing; 2 points for visible soft calculus deposits in the gum pockets and adjacent surfaces; 3 points for a large amount of soft calculus deposits in the gum pockets, with the gum margins and adjacent surfaces being 3 points.
2. The hospital-developed nursing compliance scale was used to assess the patients’ compliance with nursing procedures. This scale consisted of self-assessment (with a total score of 80 points) and other assessments (with a total score of 20 points), covering 20 items, with a total score of 100 points. Self-assessment section covered several key dimensions, including the patients’ self-evaluation of daily oral hygiene practices during orthodontic treatment, their understanding of the importance of timely follow-up visits and the actual punctuality of such visits and their self-monitoring and feedback on following the doctor’s dietary recommendations. The other assessment components were mainly conducted by healthcare professionals or members of the research team, including checking the maintenance status of the patients’ orthodontic devices, conducting professional evaluations of the patients’ oral hygiene conditions and recording the attendance and performance of the patients in orthodontic health education classes. A score of ≥90 indicated complete compliance, 75–89 indicated partial compliance, 60–74 indicated general compliance, and ≤59 indicated non-compliance. Compliance = (Number of compliant cases (completely compliant + partially compliant + generally compliant)) / Total number of cases × 100%.
3. Before the intervention and 6 months after the intervention, the Adult Health Self-Management Ability Scale was used to assess the self-management ability of patients. This scale consisted of 3 sub-scales, namely self-management behavior (14 items), self-management environment (10 items), and self-management cognition (14 items), totaling 38 items. The self-management behavior subscale mainly assessed the actual self-management actions taken by patients during orthodontic treatment. This included whether the number of times patients brush their teeth each day and the duration of each brushing meet the recommended standards; whether they use specific oral care products as prescribed by the doctor; and whether they communicate with medical staff promptly when experiencing discomfort or problems with the orthodontic devices. The self-management environment subscale focused on the influence of the environment in which the patients were located on their orthodontic self-management and their ability to adapt to and utilize the environment. This included whether the patient’s family environment supports orthodontic treatment, such as whether family members remind the patient to perform oral care on time and prepare suitable food for the patient; whether the patient’s work or study environment is conducive to oral cleaning, such as whether there are convenient facilities for washing and the working or study time allows the patient to perform oral care; and the impact of the social environment in which the patient is located on their orthodontic behavior, such as whether friends and colleagues understand and support the patient’s orthodontic treatment. The self-management cognition subscale mainly assessed the patients’ understanding and mastery of orthodontic treatment-related knowledge and their awareness of the importance of self-management. This included whether the patients understand the basic principles and processes of orthodontic treatment; whether they are clear about the key points of oral hygiene maintenance during orthodontic treatment; and whether they recognize the importance of regular follow-up visits and following medical advice for the orthodontic effect. It used a 5-point scoring system, with a total score range of 38 to 190, and the score was positively correlated with self-management ability.
4. The incidence of complications including oral infection, gingival bleeding, gingival hyperplasia, mucosal injury, and gingivitis were recorded.
5. Before the intervention and 6 months after the intervention, the Symptom Self-assessment Scale (SCL-90) was used to assess the mental health of patients (11). This scale consisted of 90 items and 9 factors. Each item was scored on a 5-point scale, with the score ranging from 1 to 5. The higher the score, the worse the patient’s mental health condition.
6. The satisfaction survey questionnaire independently compiled by the hospital was used to assess patients’ satisfaction with the nursing services. The questionnaire consists of 10 items, with a total score of 100. A score of over 85 indicated very satisfied, a score between 60 and 85 represented satisfied, and a score below 60 indicated satisfied.

In this study, the questionnaire was filled out by phone, but it was completed independently by the patients. We contacted the patients by phone. After obtaining the patients’ consent to participate in the study and their verbal informed consent, we sent the electronic questionnaire to the patients in the form of a text message link or email attachment. After receiving the questionnaire, the patients completed the filling out independently in a relatively independent and quiet environment according to their own time and convenience. To ensure that patients could complete the questionnaire smoothly, we provided detailed instructions and precautions when sending the questionnaire. If patients encountered technical problems or had difficulties understanding the questions during the filling process, they could contact our researchers at any time through the reserved contact information. The researchers promptly offered guidance and assistance. Throughout the entire process, the personal information of patients and the data from the questionnaires were strictly confidential. Only authorized researchers could access and process these data according to the prescribed procedures to safeguard the privacy of the patients.

### 2.6 Statistical analysis

To examine whether there were differences in the variables among the various groups, we conducted a quantitative analysis using SPSS 21.0 software through chi-square test, following the operational procedures based on the test conditions. All continuous data that met the normal distribution were analyzed using the student t-test, while non-parametric data were analyzed using the Mann–Whitney U test. Count data and measurement data were presented in the form of n (%) and mean ± standard deviation, respectively. All tests were two-sided. A *p* value less than 0.05 was considered to indicate a statistically significant difference.

## 3 Results

### 3.1 Baseline information

A total of 136 orthodontic patients were included in this study. There was no loss of cases between the two groups. The mean age of the patients was (29.11 ± 8.04) years, with the maximum age being 39 years and the minimum age being 17 years. There was no statistically significant difference in the baseline data between the two groups, as shown in Table 1 (*p* > 0.05).

**Table 1:** Baseline characteristics of patients (mean ± standard deviation) or *n* (%).

Parameter		Control	Intervention	*X*^2^ (*t*)	*p*
Number		68	68	-	-
Age (years)		29.71 ± 6.98	28.92 ± 7.28	0.646	0.519
Place of residence, *n* (%)	Town	42 (61.75)	47 (69.12)	0.813	0.902
	Village	26 (38.25)	21 (30.88)		
Gender, *n* (%)	Female	33 (48.53)	38 (55.88)	0.737	0.391
	Male	35 (51.47)	30 (44.12)		
Job, *n* (%)	Students	40 (58.82)	44 (64.71)	0.498	0.48
	Employment	28 (41.18)	24 (35.29)		
Matrimonial, *n* (%)	Married	25 (36.76)	21 (30.88)	0.526	0.469
	Unmarried	43 (63.24)	47 (69.12)		
Angle Classification, *n* (%)	I	24 (35.29)	20 (29.41)	1.928	0.381
	II	24 (35.29)	27 (39.71)		
	III	20 (29.41)	21 (30.88)		
Course of disease (months)		14.19 ± 3.54	13.59 ± 3.49	0.995	0.321

### 3.2 Periodontal health status

As shown in Table 2, there were no significant differences in the PLI and GI between the two groups (*p* > 0.05). At 3 months and 6 months after orthodontic treatment, the PLI and GI of the patients in both groups showed significant improvement (*p* < 0.05). Moreover, the PLI and GI of the intervention group were lower than those of the control group at 3 months and 6 months after orthodontic treatment (*p* < 0.05).

**Table 2:** Comparison of PLI and GI between the two groups of patients (mean ± standard deviation).

Items	Groups	Before intervention	3 months after intervention	6 months after intervention
PLI (points)	Control (*n* = 68)	2.41 ± 0.43	1.89 ± 0.31*	2.10 ± 0.35*
	Intervention (*n* = 68)	2.38 ± 0.51	1.68 ± 0.32*	1.81 ± 0.37*
*t*		0.370	3.886	4.695
*p*		0.711	0.000	<0.001
GI (points)	Control (*n* = 68)	2.01 ± 0.46	1.56 ± 0.36*	1.79 ± 0.41*
	Intervention (*n* = 68)	1.98 ± 0.44	1.29 ± 0.41*	1.41 ± 0.37*
*t*		0.388	4.080	5.674
*p*		0.698	0.000	<0.001

* meant compared with before intervention, *p* < 0.05.

### 3.3 Nursing compliance

**Figure 1:**
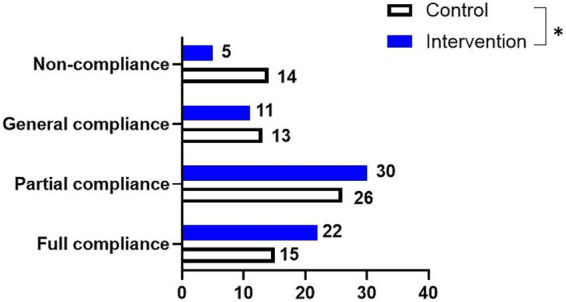
Assessment of nursing compliance in both groups. *p < 0.05.

The nursing compliance of the intervention group (92.65%) was higher than that of the control group (79.41%) (p = 0.026, Figure 1).

### 3.4 Assessment of self-management skills

**Figure 2:**
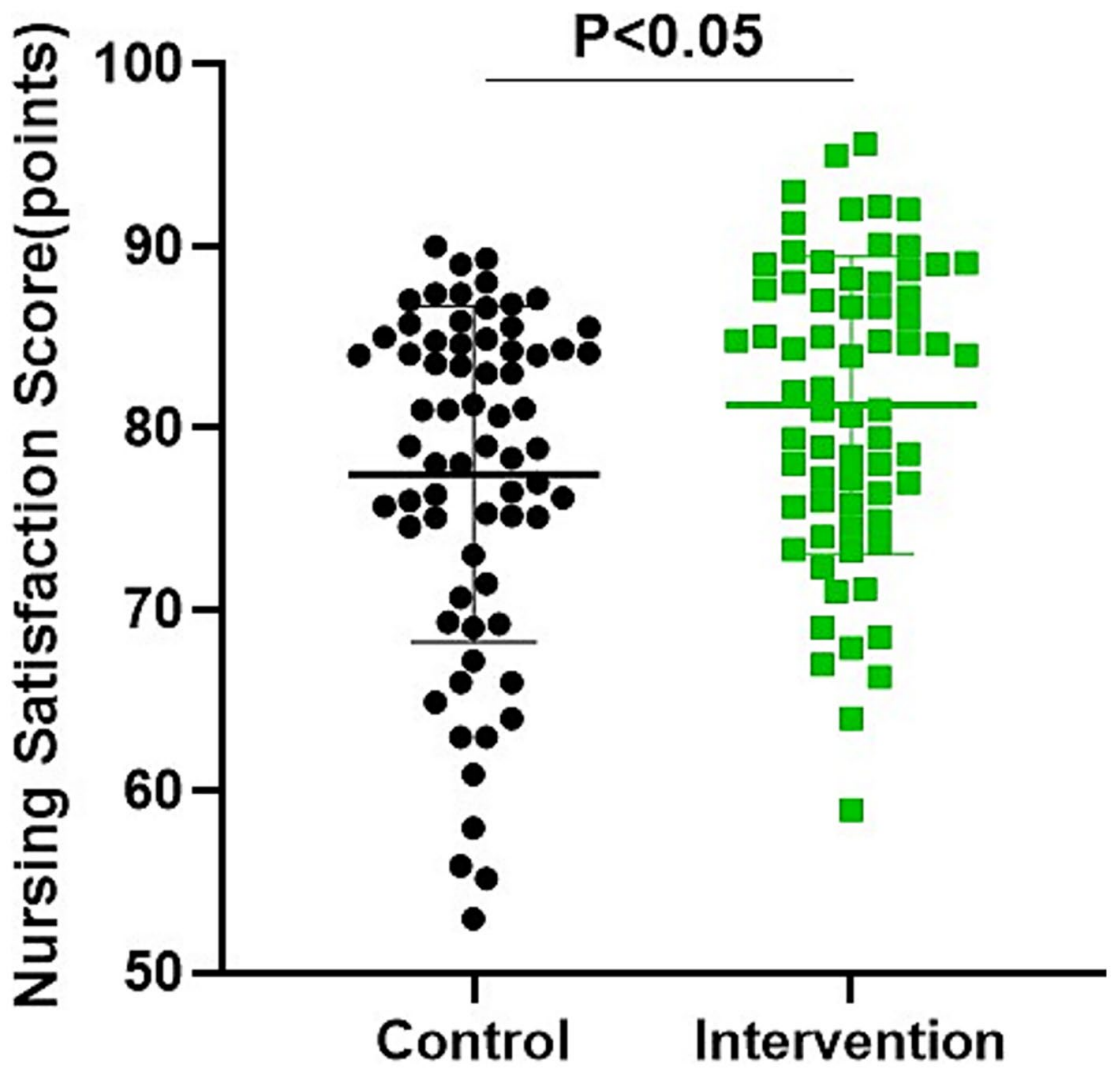
Comparison of patients’ self-management skills between the two groups. *p < 0.05, compared with control, #*p* < 0.05, compared with before treatment.

As shown in Figure 2, there were no significant differences in the scores of self-management behavior, self-management environment, and self-management cognitive between the two groups (*p* > 0.05). After 6 months of intervention, the scores of self-management behavior, self-management environment, and self-management cognitive of both groups were elevated (*p* < 0.05). Compared with the control group, the intervention group had higher scores of self-management behavior, self-management environment, and self-management cognitive (*p* < 0.05).

### 3.5 Comparison of complications between the two groups of patients

As shown in Table 3, the incidence of complications was significantly lower in the intervention group (14.71%) than that in the control group (21.00%) (*p* = 0.025), with gingival hyperplasia and gingivitis being the most common complications.

**Table 3:** Complication statistics for both groups of patients, *n* (%).

Groups	Oral infection	Bleeding gums	Gingival hyperplasia	Mucosal damage	Gingivitis	Total rates
Control (*n* = 68)	1 (1.47)	3 (4.41)	6 (8.82)	1 (1.47)	10 (14.71)	21 (30.88)
Intervention (*n* = 68)	0 (0.00)	1 (1.47)	3 (4.41)	1 (1.47)	5 (7.35)	10 (14.71)
*X* ^2^						5.056
*p*						0.025

### 3.6 Mental health assessment of patients in both groups

As shown in Table 4, compared with the control group, the intervention group had lower SCL-90 scores in terms of obsessive-compulsive disorder, interpersonal sensitivity, depression and anxiety (*p* < 0.05).

**Table 4:** Comparison of the mental health of patients in the two groups (mean ± standard deviation).

Groups	Somatisation	Obsessive-compulsive disorder	Interpersonal sensitivity	Depression	Anxiety
Control (*n* = 68)	1.36 ± 0.44	1.76 ± 0.28	1.71 ± 0.23	1.55 ± 0.36	1.61 ± 0.32
Intervention (*n* = 68)	1.39 ± 0.38	1.64 ± 0.35	1.62 ± 0.31	1.43 ± 0.33	1.48 ± 0.39
*t*	0.426	2.208	2.35	2.026	2.125
*p*	0.671	0.029	0.02	0.045	0.035

Groups	Hostile	Terror	Paranoid personality disorder	Psychotic
Control (*n* = 68)	1.49 ± 0.34	1.25 ± 0.35	1.58 ± 0.27	1.35 ± 0.25
Intervention (*n* = 68)	1.46 ± 0.31	1.21 ± 0.32	1.52 ± 0.31	1.33 ± 0.28
*t*	0.538	0.696	1.204	0.439
*p*	0.592	0.488	0.231	0.661

### 3.7 Comparison of satisfaction with nursing

**Figure 3:**
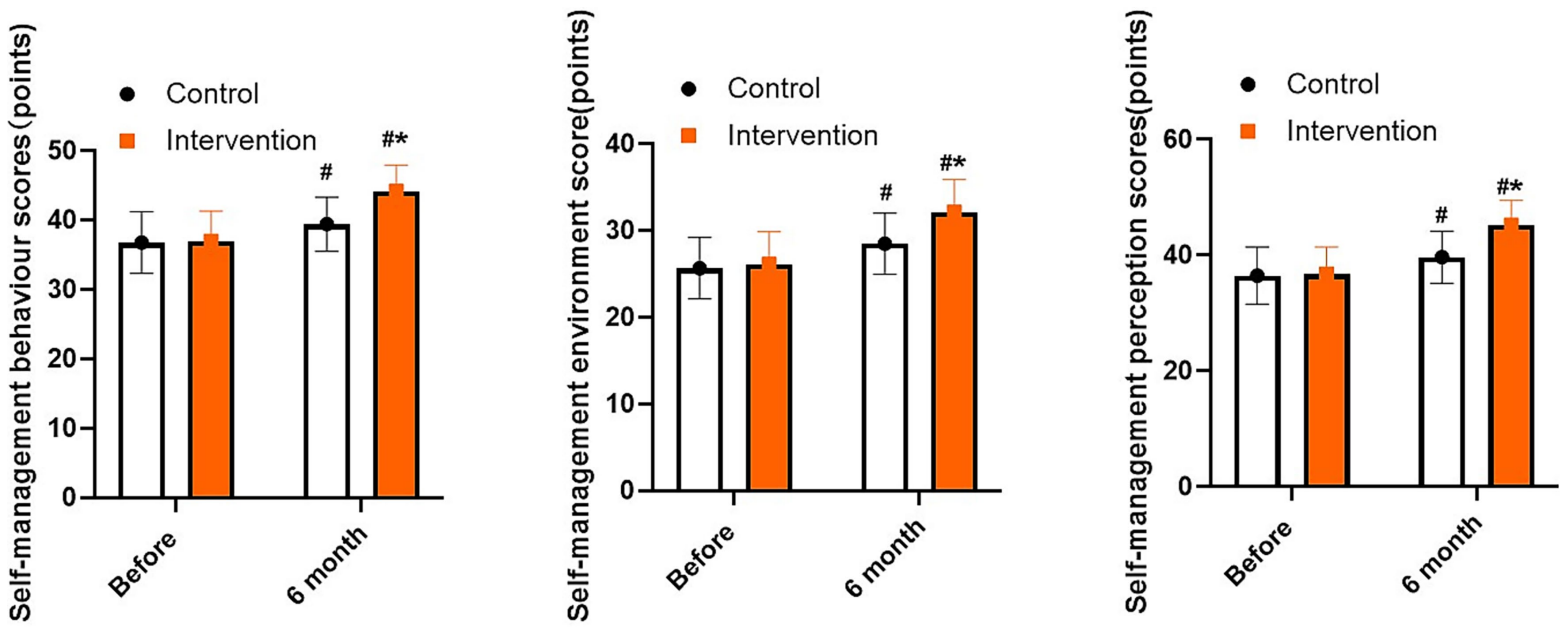
Comparison of patient care satisfaction between the two groups.

As shown in Figure 3, the satisfaction score of the intervention group was (81.25 ± 8.17) points, and that of the control group was (77.40 ± 9.35) points. Compared with the control group, the intervention group had higher satisfaction score (*p* = 0.012).

## 4 Discussion

Orthodontics is the main treatment method for improving the appearance and functionality of teeth. However, due to the long duration of orthodontic treatment, patients lack knowledge of oral health and self-management skills, which makes them prone to complications such as oral mucosal damage and plaque formation during the treatment period. This seriously affects the overall orthodontic outcome (12). Therefore, it is necessary to enhance the continuity of care for patients undergoing orthodontic treatment, improve their health education knowledge level, and strengthen their awareness and ability of oral care to reduce complications and improve the orthodontic outcome (13).

Both the PLI and GI are important indicators reflecting the condition of periodontal health. Among them, PLI is mainly used to assess the oral hygiene status, while GI is mainly used to measure the severity of gingivitis (14). This study found that at 3 months and 6 months after orthodontic treatment, the PLI and GI of the patients in both groups showed significant improvement. Moreover, the PLI and GI of the intervention group were lower than those of the control group at 3 months and 6 months after orthodontic treatment. These results suggest that continuity of care under the integrated care model can improve the periodontal health status of orthodontic patients. Plaque bacteria trigger the initial local inflammatory response, followed by the mid-stage process of immune cell migration and antibody production, and finally the active manifestation of immune cells, which leads to tissue damage and the spread of inflammation. If left untreated, the inflammation will further damage the gums and periodontal supporting tissues, causing periodontitis and gingivitis (15). Gingivitis is the most common complication during orthodontic treatment. Gingival epithelial hyperplasia is a protective mechanism of the gums to resist bacterial infections, aiming to protect the underlying tissues. The worsening of the condition is caused by the uncontrolled infiltration of inflammatory cells, bleeding of gum vessels, and the increase in matrix metalloproteinase (MMPs) activity, which eventually leads to gingivitis (16, 17). Gingivitis not only affects the daily chewing function, but also has an impact on the digestive system function. In severe cases, it can even lead to oral cancer and internal diseases (18–20). Therefore, the prevention of gingivitis is a key measure in orthodontic treatment. This study revealed that the incidence of complications in the intervention group (14.71%) was significantly lower than that in the control group (21.00%). This is because that continuity of care under the integrated care model enables the full utilization of existing medical resources. At the same time, it can provide patients with continuous, efficient and comprehensive care, emphasizing oral health education and care for patients, and meeting their various care needs. During the patient’s home care period, we still use methods such as sharing in WeChat groups and organizing health lectures to enrich the content and form of health education, providing patients with more targeted and comprehensive health education, helping patients fully master oral health knowledge, identify and correct risk factors, and ultimately guide patients to develop oral health care behaviors and habits, thereby improving gingivitis and plaque conditions during the treatment period, and improving mucosal damage situations. Consistently, Yang et al. indicated that effective nursing interventions can improve the oral condition of patients undergoing orthodontic treatment, enhance oral hygiene behaviors, and reduce symptoms of complications (21). Yuan et al. performed a systematic review and meta-analysis and indicated that continuity of care can effectively improve patients’ compliance with anticoagulant therapy, enhance their understanding of the drugs, reduce the incidence of complications and adverse events, and thereby improve the quality of life of patients (22). Choi et al. suggested that continuity of care for hypertension patients can enhance medication compliance and reduce the risks of stroke and cardiovascular diseases (23).

During orthodontic treatment, the patient’s compliance, mental health status, and other factors also play a significant role in improving the orthodontic outcome and prognosis. Throughout the treatment period, the pain and discomfort caused by the treatment, as well as the long treatment duration, can trigger negative emotions. These negative emotions can also indirectly affect the patient’s compliance with the treatment and their self-management ability, and these factors are also important indicators of the treatment outcome and rehabilitation prognosis (24). Our results suggested that after 6 months of intervention, the scores of self-management behavior, self-management environment, and self-management cognitive of both groups were elevated. Compared with the control group, the intervention group had higher scores of self-management behavior, self-management environment, and self-management cognitive. At the same time, the nursing compliance of the intervention group (92.65%) was higher than that of the control group (79.41%), and the SCL-90 scores in terms of obsessive-compulsive disorder, interpersonal sensitivity, depression and anxiety in the intervention group were lower than those in the control group. These results indicate that continuity of care under the integrated healthcare model can improve self-management, mental health and nursing compliance of orthodontic patients. Consistently, Basso et al. performed a meta-analysis and found that patients who received orthodontic surgery treatment had significant improvements in the scores of factors such as depression, obsessive-compulsive symptoms, interpersonal sensitivity, depression, and neuroticism on the SCL-90 scale (25). Lai et al. suggested that continuity of care can improve the self-management ability and quality of life of patients undergoing maintenance hemodialysis (26). Gu et al. performed a systematic review, indicating that patients with inflammatory bowel disease who can receive continuous medical care services are able to reduce the number of outpatient visits and improve medication compliance (27).

Our study has some limitations. Firstly, our sample size is relatively small, which may lead to deviations between the data results and the actual values. Second, this study is a single-center study with a relatively small sample size, which may affect the stability and reliability of the research results. Second, a single-center study may be influenced by factors such as the patient population characteristics, medical resources, and management models of a specific hospital, resulting in certain deviations in the research results. In addition, the patients included in this study span a wide age range, covering multiple age groups from teenagers to middle-aged and elderly people. There are significant differences in physiology, psychology and society among patients of different age groups, which may make it difficult for the research results to accurately reflect the true situation of patients in a specific age range. To some extent, this affects the targeted nature and generalizability of the research results. Given the limitations of the above research, future studies should carry out multi-center, large-sample randomized controlled trials. At the same time, we will further refine the age groups and carry out specialized research for orthodontic patients of different age groups. Through large-scale multi-center studies, deeply explore the differences in the effects of continuous care under the comprehensive nursing model in patients of different age groups, clarify the special needs and nursing priorities of patients of each age group, and provide a basis for formulating individualized nursing plans.

In conclusion, implementing continuity of care for orthodontic patients under the integrated care model can enhance their self-management skills, compliance, and mental health levels, reduce complications, thereby improving the treatment outcome and periodontal health of orthodontic patients, and ultimately increasing their satisfaction with the care. This care method can be promoted as an effective nursing approach for orthodontic patients.

## Data Availability

The datasets presented in this study can be found in online repositories. The names of the repository/repositories and accession number(s) can be found in the article/supplementary material.
